# Factors influencing the posterior cruciate ligament buckling phenomenon—a multiple linear regression analysis of bony and soft tissue structures of the knee joint

**DOI:** 10.1186/s13018-024-04739-3

**Published:** 2024-05-03

**Authors:** Jiaying Zhang, Tianwen Huang, Zhenyu Jia, Yangyang Yang, Tsung-Yuan Tsai, Pingyue Li

**Affiliations:** 1 Department of Graduate School, Guangzhou University of Chinese Medicine, 12 Airport Road, Guangzhou, 510405 Guangdong People’s Republic of China; 2 Guangdong Key Lab of Orthopedic Technology and Implant, General Hospital of Southern Theater Command of PLA, Guangzhou, People’s Republic of China; 3 Guangdong Key Lab of Orthopedic Technology and Implant, General Hospital of Southern Theater Command of PLA, The First School of Clinical Medicine, Southern Medical University, Guangzhou, People’s Republic of China; 4 School of Biomedical Engineering and Med-X Research Institute, Shanghai Jiao Tong University, Shanghai, People’s Republic of China; 5 Engineering Research Center for Digital Medicine of the Ministry of Education, Shanghai, People’s Republic of China

**Keywords:** ACL, PCL buckling angle, MRI

## Abstract

**Purpose:**

To determine whether posterior cruciate ligament (PCL) buckling (angular change) is associated with anterior cruciate ligament (ACL) status (intact or ruptured), meniscal bone angle (MBA), anterior tibial translation (ATT), body weight, femoral-tibial rotation (FTR), posterior tibial slope (PTS), PCL length and femoral-tibial distance (FTD) and to identify the factors that have the greatest influence.

**Methods:**

All enrolled participants were scanned with a 3.0 T, 8-channel coil MRI system (Magnetom Verio; Siemens). Bone and soft tissue parameters were measured by MIMICS software for each subject and each measured parameter was correlated with PCL buckling phenomena. The correlated and statistically significant parameters were then analyzed by multiple linear regression to determine the magnitude of the effect of the different parameters on the PCL buckling phenomenon.

**Results:**

A total of 116 subjects (50 ACL ruptured and 66 age, weight and height matched volunteers with uninjured knees) were enrolled. Among all measured parameters, there were 8 parameters that correlated with PCL angle (PCLA), of which ACL status had the strongest correlation with PCLA (r = − 0.67, *p* =  < 0.001); and 7 parameters that correlated with PCL-posterior femoral cortex angle (PCL-PCA), of which ATT had the strongest correlation with PCL-PCA (r = 0.69, *p* =  < 0.001). PCLIA was not significantly correlated with any of the measured parameters. Multiple linear regression analyses revealed four parameters can explain PCLA, of which ACL status had the strongest effect on PCLA (absolute value of standardized coefficient Beta was 0.508). Three parameters can explain PCL-PCA, of which ATT had the strongest effect on PCLIA (r = 0.69, *p* = < 0.001), ATT has the greatest effect on PCL-PCA (absolute value of normalized coefficient Beta is 0.523).

**Conclusions:**

PCLA may be a simple and easily reproducible and important supplement for the diagnosis of ACL injury; PCL-PCA is a simple and easily reproducible and important complementary tool for the detection of ATT. The use of PCLA is more recommended to aid in the diagnosis of ACL injury.

**Supplementary Information:**

The online version contains supplementary material available at 10.1186/s13018-024-04739-3.

## Introduction

The phenomenon of posterior cruciate ligament (PCL) buckling was first described in 1988 as a secondary sign on magnetic resonance imaging (MRI) after anterior cruciate ligament (ACL) injury [1, 2]. Following this, methods on quantifying the PCL buckling phenomenon have emerged. There are currently three methods for quantifying PCL buckling that are easily reproducible and have high diagnostic accuracy, including the PCL angle (PCLA) [3], the PCL inclination angle (PCLIA) [4], and the PCL-posterior femoral cortex angle (PCL-PCA) [5]. An important consequence of ACL rupture is a decrease in knee stability, which is primarily due to the loss of the ACL's ability to limit anterior tibial translation (ATT) and rotation [6]. The ACL has been shown to have a significant impact on knee stability, previous studies have shown increased anterior tibial translation (ATT) and rotational laxity of the knee after ACL rupture, and that the angle between the femoral portion (proximal horizontal portion) and the tibial portion (proximal vertical portion) of the PCL is altered [7, 8]. Both ATT and internal tibial rotation are manifestations of knee instability, probably by causing a change in the relative position of the femoral and tibial endpoints of the PCL, which results in a change in the flexion angle of the PCL.

In addition to the ACL, the structures that maintain the stability of the knee include the meniscus inside the joint as well as the ligaments and muscles that surround the joint. Studies have confirmed that posterior tibial slope (PTS) [9–11] and meniscal slope [9, 12] are risk factors for ACL injury or rupture, and also the meniscus is a secondary limiting structure for ATT [13]. Excessive PTS increases anterior tibial translation, even with ACL restriction. While the presence of a meniscus counteracts some of the PTS, different meniscus slopes counteract it differently. Among other soft tissues of the knee, an interesting recent study [14] found that patients with ACL rupture had an increased volume of the infrapatellar fat pad, but no differences were found in the volume and length of the PCL compared to controls. Whether PCL length has an effect on the phenomenon of PCL buckling is still unknown.

Up to date, the phenomenon of PCL buckling has only been used as a secondary sign on MRI after ACL injury. Although it has been suggested that ATT may be an influential factor in the alteration of PCL angle, studies on the bony and soft tissue factors that contribute to the alteration of PCL buckling angle are rare. In recent studies, PCL-PCA has been shown to be the most accurate and simple-to-repeat method for diagnosing ACL injuries [5]. And the buckling phenomenon of the PCL could be affected by the duration of the ACL injury [15]. In addition to this, other factors such as bony structures, soft-tissue structures, PCL length and demographics (e.g., body mass index (BMI), etc.) have not been included in the studies and have not been quantified.

The primary objective of this study was to determine whether other factors such as bony and soft tissue structures, in addition to the status of the ACL, influence the PCL angle; the secondary objective was to validate the determination of the magnitude of the effect of the different influences on the PCL angle. We hypothesized that ACL status is the main influence on PCL buckling angle and has the largest effect.

## Methods

### Study design

**Fig. 1:**
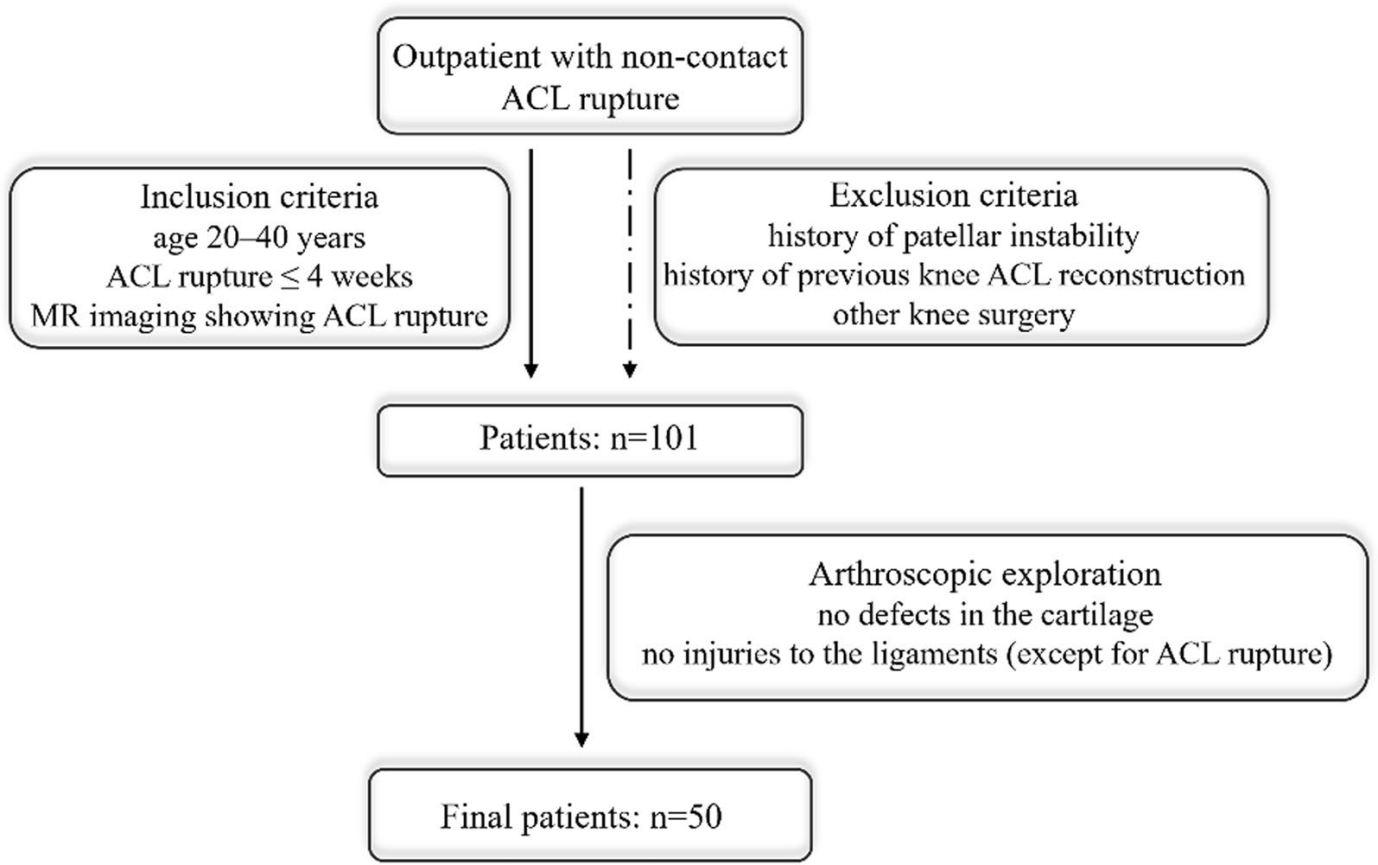
Flow chart for inclusion of patients with ACL ruptures

This cross-sectional retrospective analysis study was approved by the ethics committee. All patients with non-contact ACL ruptures seen in the outpatient setting and treated with inpatient surgery were considered potential candidates for this study. MRI records of all patients treated between May 2022 and May 2023 were retrospectively analyzed after informed consent was obtained from patients. Inclusion criteria for patients with ACL ruptures: (1) age 20–40 years, (2) ACL rupture ≤ 4 weeks from the date of presentation to admission, and (3) MR imaging showing ACL rupture. Exclusion criteria for patients with ACL ruptures: (1) history of patellar instability, and (2) history of previous knee ACL reconstruction or other knee surgery. All patients who met the inclusion and exclusion criteria had undergone MRI scan of the knee before undergoing ACL reconstruction surgery. In order to exclude the influence of other possible factors as much as possible, patients were again categorized intraoperatively and selected for arthroscopic knee joints with no defects in the cartilage of the medial and lateral compartments and no injuries to the ligaments (except for ACL rupture). To avoid subject selection bias, volunteers with healthy and uninjured knees were also included in the study. These volunteers underwent a health examination at our hospital and informed consent was obtained. None of these volunteers had uncomfortable symptoms such as ACL injury, knee pain, or a family history of OA (Fig. 1).

### Magnetic resonance protocols

All enrolled participants who obtained informed consent were scanned with a 3.0 T, 8-channel coil MRI system (Magnetom Verio; Siemens): proton weighted (repetition time [TR], 1300 ms; echo time [TE], 38 ms; slice thickness, 0.5 mm; field of view, 100 mm; flip Angle, 180°) in the supine position with the knee in extension and a sandbag placed on the knee to stabilize the knee and prevent any motion artifacts.

### MRI measurement method

**Fig. 2:**
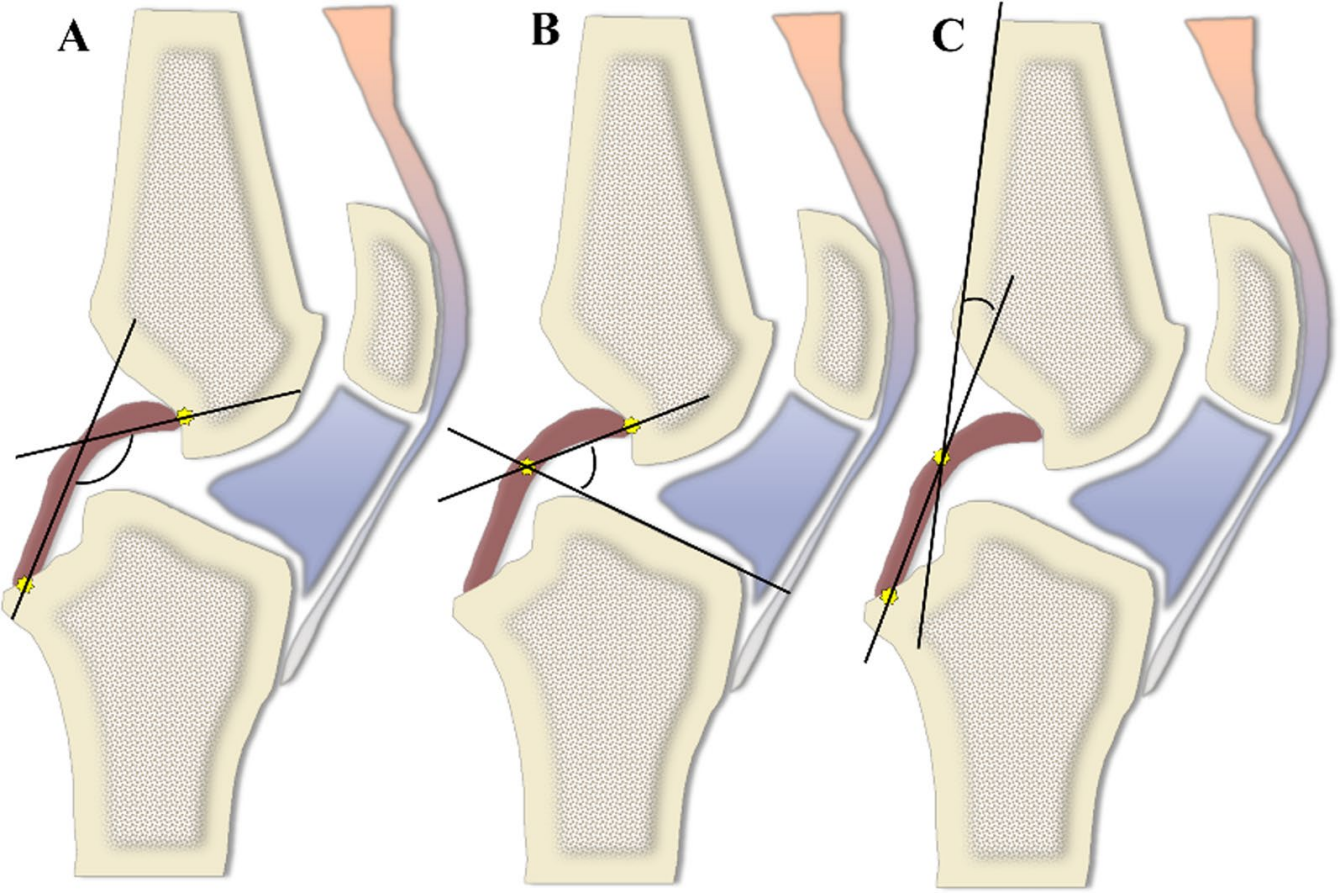
**A** PCLA: was defined as the angle between a line through the center portion of the tibial endpoint of the PCL (and parallel to the lower portion of the PCL) and a line through the center portion of the femoral endpoint of the PCL (and parallel to the upper portion of the PCL). **B** PCLIA: one is the tangent line to the articular surface of the tibial plateau and intersects the PCL, and the other is the line connecting the center of the femoral endpoint of the PCL to the intersection between the first line and the PCL. **C** PCL—PCA: one line parallels the posterior cortex of the femoral stem and the other line parallels the tibial portion of the PCL alignment

**Fig. 3:**
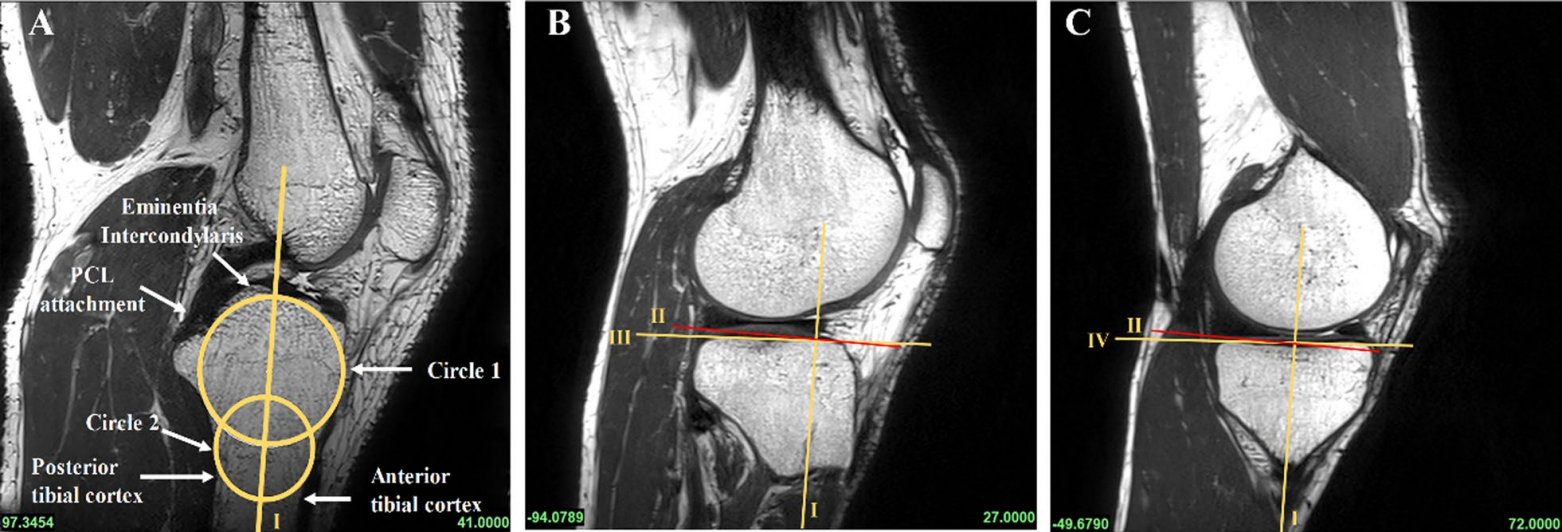
A central sagittal image that clearly shows the attachment point of the PCL and the intercondylar eminence is selected and two circles are drawn on the proximal tibia (**A**). Circle 1 is tangent to the tip of the tibia and the anterior and posterior tibial cortex, and circle 2 is tangent to the anterior and posterior tibial cortex, with the center of the circles located on the circumference of circle 1. The centers of the two circles were connected as the anatomical axis of the tibia (**A**). Tangent lines to the medial and lateral tibial plateaus are then made on the central sagittal image. The angle between the tangent lines of the medial and lateral tibial plateaus and the vertical line (red line) of the anatomical axis of the tibia is the medial PTS (MPTS) (**C**) and lateral PTS (LPTS) (**B**)

**Fig. 4:**
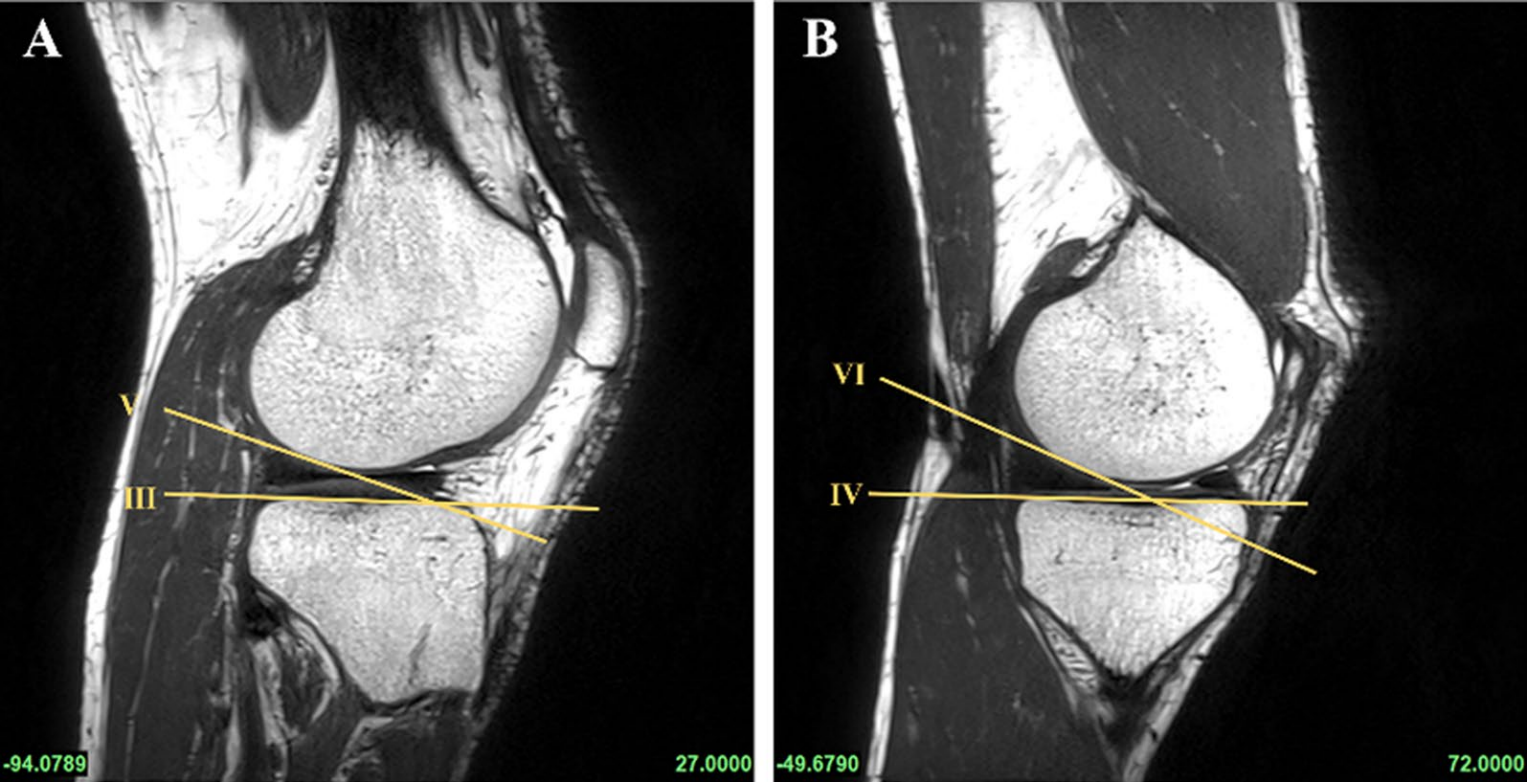
Draw a line from the highest and proximal points of the posterior horn of the meniscus and deliver it forward by the most meniscus superior surface (Lines V and VI). The angle between the tangent lines of the medial and lateral tibial plateaus and the drawn line V and VI was defined as the MBA. Superimposing the MBA and the corresponding PTS, we obtained the lateral combined slope (LCS) and medical combined slope (MCS) angles. **A** lateral MBA; **B** medial MBA

**Fig. 5:**
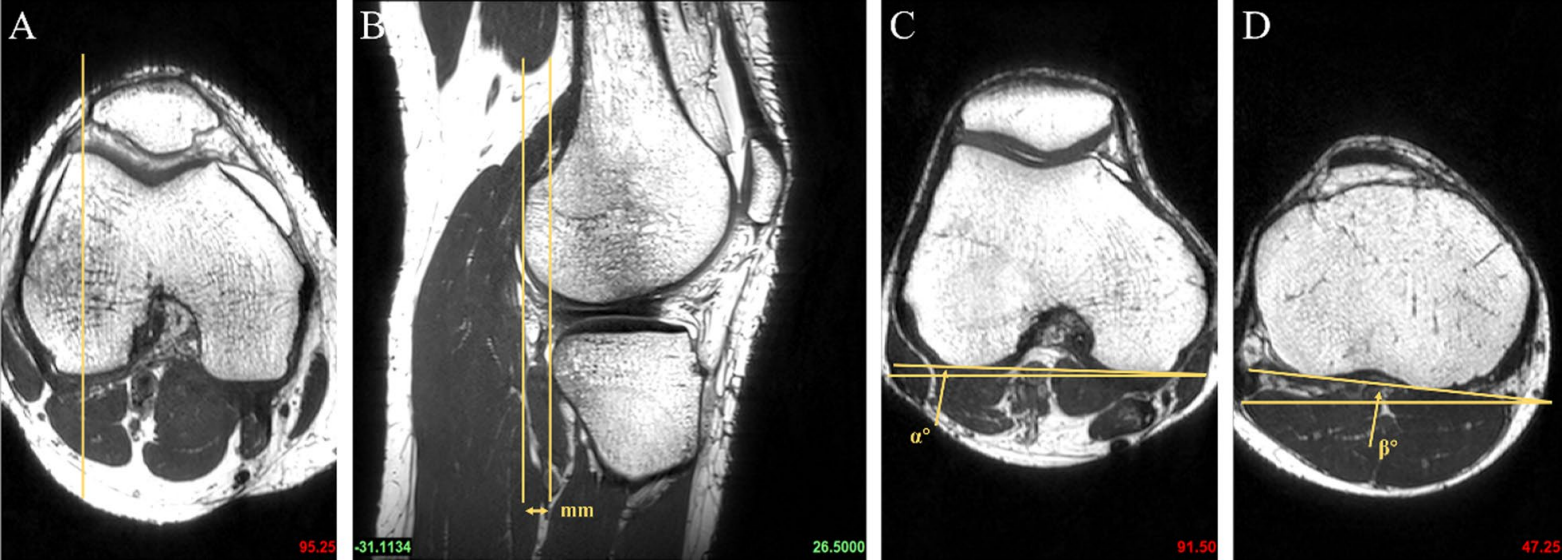
The midpoint of the lateral femoral condyle was identified on the axial MRI image (**A**). Two parallel lines tangent to the posterior cortex of the lateral femoral condyle and the posterior tibial cortex are made on the corresponding sagittal image (**B**). The horizontal distance between these two lines is the ATT (unit: millimeter) (if the tibial posterior cortex tangent line is located before the femoral posterior cortex tangent line, it is recorded as negative, indicating anterior tibial displacement; vice versa, it is positive). The widest MRI image level in the anteroposterior direction of the femoral condyles was first determined and then determine the first axial MRI image above the fibular head. The angle between the tangent line of the posterior cortex of the femoral condyle and the horizontal line was defined as the femoral angle (**C**). The angle between the posterior cortical tangent of the tibial condyle and the horizontal line was defined as the tibial angle (**D**). Internal rotation was considered positive and external rotation negative. The FTR was obtained by measuring the absolute difference between these two angles

**Fig. 6:**
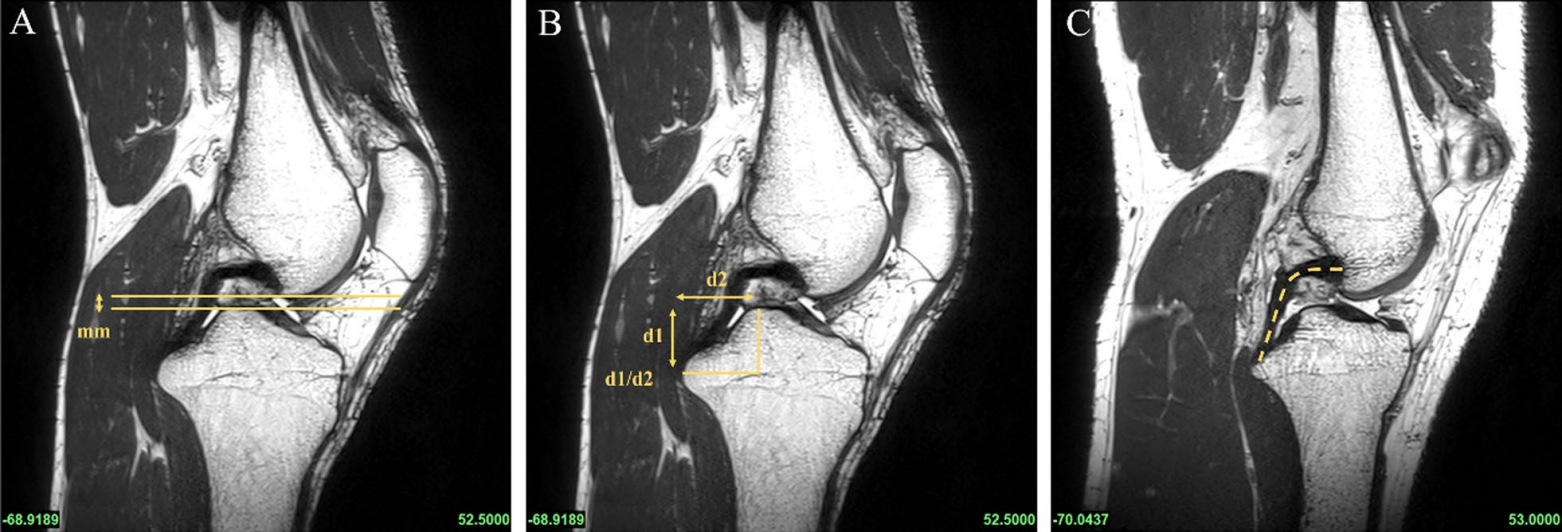
On the sagittal image, which shows the PCL most clearly (**A**–**C**), a horizontal line is drawn through the lowest point of the femur and the highest point of the tibia, and the distance between the two lines is the FTD (**A**). d1 represents the vertical distance from the intercondylar eminence to the PCL tibial endpoint, and d2 represents the horizontal distance from the intercondylar eminence to the PCL tibial endpoint; the ratio of d1–d2 was defined as IER (**B**). The length of the PCL was defined as the maximum length of the sagittal plane showing the clearest PCL; a line was drawn from the center of the femoral endpoint of the PCL, along the center of the PCL alignment, to the center of the tibial endpoint of the PCL (**C**)

According to previous methods in the literature, all subjects underwent PCL buckling angle measurements on MRI sagittal images, including three methods: PCLA, PCLIA, and PCL-PCA (PCLA and PCLIA were measured on the sagittal plane of the MRI showing the clearest PCL, and PCL-PCA was measured on the sagittal image of the most lateral portion of the tibial endpoint of the PCL) (Fig. 2). PTS (lateral and medial PTS were measured separately): on MRI sagittal images, it was measured according to the method of Hudek et al. [16] (Fig. 3). The PTS angle was defined as posterior (-) if the plateau tangent lines was inferior to the perpendicular line and anterior (+) if the plateau line was superior to the perpendicular line (the smaller the value, the greater the slope). Meniscal bone angle (MBA) (lateral and medial MBA were measured separately): on MRI sagittal images, it was measured according to the method of Hohmann et al. [12] and Bojicic et al. [17] (Fig. 4). ATT: Measured on MRI sagittal images according to the method described in the literature [18, 19] (Fig. 5). Femoral tibial rotation (FTR) angle: according to the method of Vassalou et al. [20] (Fig. 5). Femoral-tibial distance (FTD), Intercondylar eminence ratio (IER) and PCL length were shown in Fig. 6. PCL length was measured according to the method of Fontanella et al. [14]

### Reliability analysis

All MRI measurement levels were determined by a radiologist with 15 years of experience. Measurements of all parameters in the selected images were performed by two experienced orthopedic surgeons. Intra- and inter-observer reliability of the two orthopedic surgeons who performed the parameter measurements was assessed using intragroup correlation coefficients (ICC).

### Sample size estimation

Sample size estimates for multiple linear regression analysis using PASS software (version 15.0, NCSS, LLC); efficacy: 0.8. alpha: 0.05. Regression Model Type: Unconditional (Random X's). Kc (Number of X's Controlled): 0, Kt (Number of X's Tested): 9, f^2^: 0.15. A sample size of 116 achieves 80% power to detect an effect size (f^2^) of 0.150 attributable to 9 independent variable(s) using an F-Test with a significance level (alpha) of 0.05. The calculations assume an unconditional (random X's) model.

### Statistical analysis

Statistical analysis was performed using SPSS software (IBM SPSS Statistics for Windows, Version 26.0. Armonk, NY: IBM Corp). The Kolmogorov–Smirnov test was used for the normal distribution of continuous variables. The independent samples t-test or Mann–Whitney U-test was used as determined by the normality test. The independent samples t-test results are presented as mean and standard deviation (SD), whereas the Mann–Whitney U-test results are presented as median and interquartile range (IQR). Intra- and inter-observer reliability was assessed using intragroup correlation coefficient (ICC) (absolutely consistent two-way mixed single measure) and classified as slight (0–0.2), fair (0.21–0.4), moderate (0.41–0.6), good (0.61–0.8) and excellent (> 0.8). Correlations between PCL flexion angle and each parameter were assessed using Spearman correlation coefficients for categorical variables and Pearson correlation coefficients for continuous variable parameters (|r|< 0.3, weak correlated; 0.3 <|r|< 0.5, low correlation; 0.5 <|r|< 0.8, significant correlation; 0.8 <|r|< 1, highly correlated). Multiple linear regression analysis (stepwise method) was used to determine the relationship between each parameter and PCL flexion angle. A *p* < 0.05 was considered a statistically significant difference.

## Results

### Participants characteristics

Based on the results of arthroscopic exploration, inclusion and exclusion criteria, 50 patients with ACL rupture were finally enrolled in the study; 66 healthy volunteers matched for age, weight and height were included in the study (66 healthy volunteers were selected primarily to fulfill the 80% power of the test and secondarily to match the male-to-female ratio to the ACL rupture group as much as possible). Finally, a total of 116 subjects were included (subjects baseline characteristics were shown in Table 1). The arthroscopic findings were shown in Additional file 1: Table S1 (arthroscopic findings in all patients with ACL rupture showed smooth and undamaged cartilage surfaces).

**Table 1:** Baseline characteristics of subjects (means ± sd)

Group	Male	Female	Age (y)	Weight (kg)	Height (m)
Uninjured	60	6	25.06 ± 2.04	73.76 ± 12.32	1.73 ± 6.30
Injured	46	4	26.31 ± 6.52	75.69 ± 11.01	1.72 ± 8.05
*p* value	–	–	0.15	0.38	0.53

### Reliability

The inter- and intra-observer ICC for two observers who performed the parameters were greater than 0.9 (Additional file 1: Table S2).

### Parameter differences between ACL injured and uninjured

After analyzing all the parameters in both the groups, MPTS, FTD, IER (Table 2) as well as PCLIA (Table 3) were not found to be statistically different between the two groups. Among the parameters that were statistically significant between the groups, LPTS, LMBA, MMBA, LCS, MCS and ATT were smaller and FTR was larger in the ACL rupture group than in the control group. This result indicates that the slope of bony and soft tissue structures in the ACL rupture group was more posteriorly inclined, with more pronounced anterior tibial translation and relative femoral rotation. With regard to the PCL buckling phenomenon, the PCL angle was smaller in the ACL rupture group (both PCLA and PCL-PCA), indicating more severe PCL buckling.

**Table 2:** Differences in bone and soft tissue parameters between Injured and Uninjured groups (mean ± sd)

Parameters	Injured	Uninjured	*p* value
LPTS	− 3.31 ± 4.06	− 1.67 ± 3.84	**0.03**
MPTS	− 3.97 ± 3.33	− 3.36 ± 3.11	0.33
LMBA	22.25 ± 6.46	26.72 ± 5.01	**< 0.001**
MMBA	23.59 ± 4.87	26.59 ± 3.51	**< 0.001**
LCS	18.94 ± 6.84	25.05 ± 6.05	**< 0.001**
MCS	19.62 ± 5.64	23.23 ± 4.20	**< 0.001**
ATT	− 4.72 ± 4.24	0.47 ± 3.36	**< 0.001**
FTD	5.87 ± 1.69	5.70 ± 2.45	0.69
FTR	11.69 ± 6.41	8.88 ± 4.38	**0.01**
IER	0.92 ± 0.14	0.92 ± 0.13	0.93
PCL length	43.98 ± 3.10	42.01 ± 3.21	**0.002**

Differences in bony and soft tissue structure between ACL ruptures and healthy controls are demonstrated in the table, *p* value less than 0.05 have been given in boldface in the table

**Table 3:** Difference in degrees of PCL buckling angle between Injured and Uninjured groups (mean ± sd)

Parameters	Injured	Uninjured	P-value
PCLA	124.39 ± 8.51	139.49 ± 8.96	**< 0.001**
PCLIA	40.43 ± 4.56	42.27 ± 5.35	0.06
PCL-PCA	25.73 ± 7.25	35.86 ± 7.68	**< 0.001**

Differences in PCL buckling angle between ACL ruptures and healthy controls are shown in the table, *p* value less than 0.05 have been given in boldface in the table

### Correlation

Of all the parameters measured, eight parameters had correlations with PCLA (Table 4), with six parameters having correlation coefficients r with absolute values greater than 0.3, including ACL status (r = − 0.67, *p* =  < 0.001), ATT (r = 0.50, *p* =  < 0.001), LCS (r = 0.41, *p* =  < 0.001), BMI (r = − 0.40, *p* =  < 0.001), LMBA (r = 0.38, *p* =  < 0.001) and MCS (r = 0.37, *p* =  < 0.001). There were seven parameters that correlated with PCL-PCA (Table 1), five of which had correlation coefficients r with absolute values greater than 0.3, including ATT (r = 0.69, *p* =  < 0.001), ACL status (r = − 0.56, *p* =  < 0.001), BMI (r = − 0.38, *p* =  < 0.001), LMBA (r = 0.35, *p* =  < 0.001) and LCS (r = 0.35, *p* =  < 0.001). PCLIA was not significantly correlated with any of the measured parameters.

**Table 4:** Correlation results between different PCL angles and different parameters

	PCLA		PCLIA		PCL-PCA	
	r	*p*	r	*p*	r	*p*
BMI	**− 0.40**	**< 0.001**	− 0.14	0.15	**− 0.38**	**< 0.001**
ACL status	**− 0.67**	**< 0.001**	− 0.17	0.07	**− 0.56**	**< 0.001**
LPTS	0.16	0.10	0.03	0.72	0.10	0.31
MPTS	**0.25**	**0.01**	− 0.07	0.44	0.15	0.13
LMBA	**0.38**	**< 0.001**	− 0.16	0.09	**0.35**	**< 0.001**
MMBA	**0.25**	**0.01**	− 0.08	0.41	0.13	0.18
LCS	**0.41**	**< 0.001**	− 0.12	0.22	**0.35**	**< 0.001**
MCS	**0.37**	**< 0.001**	− 0.11	0.24	**0.20**	**0.04**
ATT	**0.50**	**< 0.001**	0.003	0.98	**0.69**	**< 0.001**
FTD	− 0.10	0.29	− 0.09	0.33	− 0.16	0.09
FTR	− 0.08	0.40	− 0.14	0.13	− 0.11	0.26
IER	− 0.15	0.11	− 0.10	0.28	**− 0.24**	**0.01**
PCL length	− 0.18	0.06	− 0.17	0.08	− 0.18	0.07

Parameters that are correlated and statistically significant (*p* value less than 0.05) have been given in boldface in the table

### Multiple linear regression results

Multiple linear regression analyses (stepwise method) were performed respectively on the parameters that had correlations with PCLA and PCL-PCA, and the results showed that four parameters can explain PCLA, including ACL status, BMI, MPTS, and LMS, in which ACL status had the greatest effect on PCLA (absolute value of standardized coefficient of Beta was 0.508) (Table 5). There are three parameters that can account for PCL-PCA, including ATT, ACL status, and IER, with the ATT having the greatest effect on PCL-PCA (absolute value of normalized coefficient Beta is 0.523) (Table 6) (The representations of the different variable parameters and their types are shown in Additional file 1: Table S3).

**Table 5:** Results of multiple linear regression analysis of PCLA

	B	Beta	t	*p*	F	Adjusted *R*^2^
ACL status	− 11.823	− 0.508	− 6.336	< 0.001	26.406^***^	0.480
BMI	− 0.639	− 0.178	− 2.380	0.019		
MPTS	0.609	0.170	2.440	0.016		
LCS	0.286	0.150	2.015	0.046		

Beta represents the standardized regression coefficient in a multiple linear regression equation. The larger the absolute value of Beta, the greater the effect on the dependent variable. Asterisks represent *p* < 0.001

**Table 6:** Results of multiple linear regression analysis of PCL-PCA

	B	Beta	t	*p*	F	Adjusted *R*^2^
ATT	1.041	0.523	6.640	< 0.001	45.226^***^	0.547
ACL status	− 4.761	− 0.261	− 3.339	0.001		
IER	− 13.463	− 0.197	− 3.034	0.003		

Beta represents the standardized regression coefficient in a multiple linear regression equation. The larger the absolute value of Beta, the greater the effect on the dependent variable. Asterisks represent *p* < 0.001

## Discussion

The most important findings of this study were that ACL status had the greatest effect on PCLA (absolute value of standardized coefficient Beta was 0.508) and ATT had the greatest effect on PCL-PCA (absolute value of standardized coefficient Beta was 0.523). A secondary finding was that BMI, as well as a number of other bony and soft tissue structures, had an effect on both PCLA and PCL-PCA. The present findings suggest that PCLA may be a simple and easily reproducible and important supplement for the diagnosis of ACL injury; PCL-PCA is a simple and important supplement for the diagnosis of ATT.

Since the phenomenon of PCL flexion on imaging of ACL-injured individuals was first described in 1988, different methods of quantifying the PCL buckling phenomenon have emerged successively in the following decades. Simple and easily reproducible measurements include PCLA described by McCauley et al. [3]. PCLIA described by Gali et al. [4] and PCL-PCA described by Siboni et al. [5]. These three methods allow the clinical orthopedic surgeon to make simple and quick judgments about the patient. Although there are more methods to quantify the PCL buckling phenomenon, there are only a few studies on the factors influencing the PCL buckling phenomenon. A recent study by Oronowicz et al [15] found that the size of the PCL-PCA correlated with the duration of ACL injury and did not correlate with the PTS and whether the meniscus was torn. Unfortunately, in the study by Oronowicz et al., the PTS was measured on X-plain radiographs, which did not allow for differentiation between medial and lateral PTS; and only whether the meniscus was torn or not was considered, not the size of the meniscal slope.

Despite the role of the ACL in limiting anterior tibial translation, greater PTS increases the amount of anterior tibial translation has been demonstrated in studies [21, 22]. Even in populations with intact ACLs, medial and lateral PTSs have great differences. Therefore, PTS in different knee compartments (includes lateral PTS and medial PTS) should be taken into account. In the current study, only a weak correlation was found between MPTS and PCLA, and multiple linear regression analyses also showed that MPTS poorly explained PCLA (Beta absolute value of the standardized correlation coefficient was 0.17). The meniscus is the secondary important structure limiting anterior tibial translation [13] and the posterior horn of the meniscus is higher than the anterior horn, so the presence of the meniscus (especially the posterior horn of the meniscus) counteracts a portion of the PTS [23]. Therefore, in the current study, we not only measured the medial and lateral meniscal bone angles [12, 17] but also calculated the medial and lateral combined slope angles separately to quantify the extent to which the meniscus counteracted the PTS. We found that LMBA, MMBA, LCS, and MCS were all correlated with PCLA, but after multiple linear regression analysis only LCS was found to have some degree of explanatory power for PCLA (Beta absolute value of standardized correlation coefficient was 0.15). In addition to ATT, we measured the relationship between rotational laxity of the knee and the buckling angle of the PCL. Studies have shown that femoral-tibial internal rotation (FTR) can cause a change in the relative position of the femoral and tibial stops of the PCL, which can lead to a change in the flexion angle of the PCL [7, 8]. However, in our study, we did not find any correlation between FTR and PCL buckling.

Previous studies have found that BMI may affect the thickness of subcutaneous soft tissues [24]. This is because the greater the BMI, the relatively greater the body weight and the greater the load carried, which affects the thickness of subcutaneous soft tissues. This possibility was only mentioned in the study by Oronowicz et al. without a correlation analysis of whether BMI was related to PCL buckling angle. In the current study, we measured two parameters, BMI and FTD. FTD is the vertical distance from the most distal end of the femur to the most proximal end of the tibia on the most clearly sagittal MRI image shown at the PCL, and is used to reflect the magnitude of the thickness of the subcutaneous tissue. We found that BMI was significantly negatively correlated with PCLA as well as PCL-PCA, whereas FTD did not correlate with the two above mentioned PCL angles (including PCLA and PCL-PCA); this result suggests that the larger the BMI, the more severe the PCL buckling phenomenon. However, after multiple linear regression analysis, it was found that BMI had a certain degree of explanatory power only for PCLA (Beta absolute value of standardized correlation coefficient was 0.178).

In the current study, we also considered anatomical factors. The intercondylar eminence is present above the tibial endpoint of the PCL, and the alignment of the near-vertical portion of the PCL is approximately parallel to the line from the intercondylar eminence to the tibial endpoint of the PCL. We therefore measured the intercondylar eminence height (vertical distance of the intercondylar eminence from the tibial endpoint of the PCL) and width (horizontal distance of the intercondylar eminence from the tibial endpoint of the PCL) and calculated the intercondylar eminence ratio (intercondylar eminence height/ intercondylar eminence width) as a result of normalization. We found a negative correlation between IER and PCL-PCA, indicating that the higher the intercondylar eminence, the smaller the PCL-PCA angle, and the more perpendicular the tibial portion of the PCL alignment. PCL length was also considered in the current study according to Fontanella et al [14]. We found that the PCL length was greater in the ruptured ACL group compared to the healthy control group. Although the PCL length between the groups was statistically significant, the differences were very small.

Although more bony and soft tissue parameters were addressed in the current study, there are still limitations. First, for subcutaneous soft tissue thickness measurements were performed in the supine state with the knee in the straight position, and the next study should be performed in the weight-bearing position. Although changes in body position affect subcutaneous soft tissue thickness, we found a correlation between BMI and PCL buckling angle, and measurements in the weight-bearing position in subsequent studies could better complement this result. Secondly, regarding the measurement of IER, our current measurements are simple measurements based on anatomical structures, and the next step should be to incorporate more anatomical structures for measurement.

## Conclusions

PCLA may be a simple and easily reproducible and important supplement for the diagnosis of ACL injury; PCL-PCA is a simple and easily reproducible and important complementary tool for the detection of ATT. The use of PCLA is more recommended to aid in the diagnosis of ACL injury.

### Supplementary Information

**Additional file 1: **Results of the arthroscopic exploration.

## Data Availability

The datasets generated and/or analyzed during the current study are not publicly available due to the occupational characteristics of the subjects, but are available from the corresponding author on reasonable request.

## Abbreviations

PCL: Posterior cruciate ligament
MRI: Magnetic resonance imaging
ACL: Anterior cruciate ligament
PCLA: PCL angle
PCLIA: PCL inclination angle
PCL-PCA: PCL-posterior femoral cortex angle
ATT: Anterior tibial translation
PTS: Posterior tibial slope
LPTS: Lateral posterior tibial slope
MPTS: Medial posterior tibial slope
BMI: Body mass index
MBA: Meniscal bone angle
LMBA: Lateral meniscal bone angle
MMBA: Medial meniscal bone angle
FTP: Femoral tibial rotation
FTD: Femoral-tibial distance
IER: Intercondylar eminence ratio
LCS: Lateral combined slope
MCS: Medical combined slope
ICC: Intragroup correlation coefficients
SD: Standard deviation
IQR: Interquartile range
