# Supplementary material for: Factors influencing the posterior cruciate ligament buckling phenomenon—a multiple linear regression analysis of bony and soft tissue structures of the knee joint
Source: J Orthop Surg Res. 2024 May 3;19:277. doi: 10.1186/s13018-024-04739-3 (PMC11067078; doi:10.1186/s13018-024-04739-3)
Supplement: Supplementary file 1 — Additional file 1: Results of the arthroscopic exploration. [file 13018_2024_4739_MOESM1_ESM.docx]

Table S1

Results of the arthroscopic exploration

| Tissue | Compartment | Lateral | | Medial | |
| --- | --- | --- | --- | --- | --- |
|  |  | N | % | N | % |
| Cartilage | Femur | 0 | 0 | 0 | 0 |
|  | Tibial | 0 | 0 | 0 | 0 |
|  |  |  |  |  |  |
| Meniscus | Anterior horn | 1 | 2 | 0 | 0 |
| abnormalities | Corpus | 7 | 14 | 3 | 6 |
| location | Posterior horn | 21 | 42 | 15 | 28 |
|  | Other | 0 | 0 | 0 | 0 |
|  |  |  |  |  |  |
| Meniscal tear | Horizontal | 1 | 2 | 0 | 0 |
|  | Radial/vertical | 19 | 39 | 15 | 30 |
|  | Bucket-handle | 2 | 4 | 3 | 6 |
|  | Ramp | 1 | 2 | 0 | 0 |
|  | Complex | 4 | 8 | 0 | 0 |
|  |  |  |  |  |  |
| Other | Popliteal tendon injury | 1 | 2 | 0 | 0 |

Table S2

Inter- and intra-reliability Rated Intraclass Correlation Coefficient (ICC) of Two Observers

| Parameters | ICC | 95% confidence interval | Agreement |
| --- | --- | --- | --- |
| PCLA | 0.994 | 0.991-0.996 | Excellent |
| PCLIA | 0.991 | 0.986-0.994 | Excellent |
| PCL-PCA | 0.997 | 0.995-0.998 | Excellent |
| LPTS | 0.997 | 0.996-0.997 | Excellent |
| MPTS | 0.996 | 0.996-0.997 | Excellent |
| LMBA | 0.994 | 0.993-0.995 | Excellent |
| MMBA | 0.972 | 0.967-0.977 | Excellent |
| ATT | 0.993 | 0.990-0.995 | Excellent |
| FTD | 0.808 | 0.733-0.864 | Excellent |
| FTA | 0.996 | 0.994-0.997 | Excellent |
| IER | 0.989 | 0.984-0.993 | Excellent |
| PCL length | 0.961 | 0.942-0.974 | Excellent |

Table S3

Representation of different variable parameters and their types

| Variables |  | Meaning |  | Variable assignment |
| --- | --- | --- | --- | --- |
| BMI |  | body mass index |  | continuous variable (kg/m^2^) |
| ACL status |  | ACL intact or broken |  | categorical variable (1: intact, 2: broken) |
| LPTS |  | lateral posterior slope of tibial plateau |  | continuous variable (^o^) |
| MPTS |  | medial posterior slope of tibial plateau |  | continuous variable (^o^) |
| LMBA |  | lateral meniscal bone angle |  | continuous variable (^o^) |
| MMBA |  | medial meniscal bone angle |  | continuous variable (^o^) |
| LCS |  | lateral combined slope |  | continuous variable (^o^) |
| MCS |  | medial combined slope |  | continuous variable (^o^) |
| ATT |  | anterior tibial translation |  | continuous variable (mm) |
| FTD |  | Femorotibial distance |  | continuous variable (mm) |
| FTA |  | Femorotibial angle |  | continuous variable (^o^) |
| IER |  | Intercondylar eminence ratio |  | continuous variable |
| PCL length |  | Posterior cruciate ligament length |  | continuous variable (mm) |
